# Assessment of log‐based fingerprinting system of Mobius3D with Elekta linear accelerators

**DOI:** 10.1002/acm2.13480

**Published:** 2021-11-27

**Authors:** Yu‐Yun Noh, Jihun Kim, Jin Sung Kim, Han‐Back Shin, Min Cheol Han, Tae Suk Suh

**Affiliations:** ^1^ Department of Biomedical Engineering and Research Institute of Biomedical Engineering College of Medicine The Catholic University of Korea Seoul South Korea; ^2^ Department of Radiation Oncology Yonsei Cancer Center College of Medicine Yonsei University Seoul South Korea; ^3^ Department of Radiation Oncology Yonsei University College of Medicine Seoul South Korea

**Keywords:** Elekta LINAC, matching error, Mobius3D, patient‐specific QA

## Abstract

**Purpose:**

The purpose of this study was to investigate the matching error that occurs when the Mobius3D fingerprinting system is applied in conjunction with an Elekta linear accelerator (LINAC) and to offer an acceptable and alternative method for circumventing this problem.

**Material and methods:**

To avoid the multileaf collimator (MLC) conflicting error in the Mobius3D fingerprinting system, we developed an in‐house program to move the MLC in the Digital Imaging and Communications in Medicine (DICOM) radiotherapy (RT)‐Plan to pertinent positions, considering the relationship between log data and planned data. The re‐delivered log files were calculated in the Mobius3D system, and the results were compared with those of corrected data (i.e., we analyzed a pair of re‐collected log data and the previous DICOM RT‐Plan data). The results were then evaluated by comparing several items, such as point dose errors, gamma index (GI) passing rates, and MLC root‐mean‐square (RMS) values.

**Results:**

For the point dose error, the maximum difference found was below 2.0%. In the case of GI analysis of all plans, the maximum difference in the passing rates was below 1.4%. The statistical results obtained using a paired Student's *t*‐test showed that there were no significant differences within the uncertainty. In the case of the RMS test, the maximum difference found was approximately 0.08 mm.

**Conclusions:**

Our results showed that all the mismatched log files were sufficiently acceptable within the uncertainty. We conclude that the matching error obtained when applying Mobius3D to an Elekta LINAC may be addressed using a simple modification of the fingerprinting system, and we expect that our study findings will help vendors resolve this issue in the near future.

## 1 INTRODUCTION

Intensity‐modulated radiation therapy (IMRT) is an optimized planning technique that maximizes the dose to the target and minimizes the dose to the normal tissue using a multileaf collimator (MLC) and multiple segments of each beam.^1^ The IMRT technique comprises a complex process involving MLC movement, various dose rates, and gantry rotation variations; hence, this technique requires patient‐specific quality assurance (QA) for comprehensive IMRT dose verification, and especially, to confirm the correct delivery of steep dose gradient regions.^2^, ^3^, ^4^ The implementation of IMRT QA procedures before a patient receives radiotherapy is strongly encouraged by the American Association of Physicists in Medicine, the American Society for Radiation Oncology, and the American College of Radiology.^5^, ^6^, ^7^, ^8^, ^9^

In clinical practice, conventional methods for patient‐specific QA in IMRT require performing phantom‐based QA using ion chambers, films, and diode arrays.^10^, ^11^, ^12^ These procedures include the creation of a QA‐dedicated plan based on a phantom, delivering the plans to the phantom, and analyzing the measurement data (e.g., point dose(s), and 2D dose distribution(s)) in comparison with the planned data. Unfortunately, the aforementioned method could potentially suffer from three major inconveniences: (1) it could take a long time and much effort to create a phantom‐based plan for patient‐specific QA and set‐up a phantom for measurement; (2) the QA‐dedicated phantom could be unrepresentative of the inhomogeneity of patient‐specific anatomy; and (3) the 2D dose distribution and its related gamma analysis could be insufficient for verifying the complicated IMRT plan.

Recently, a new pre‐treatment QA method based on machine log data has been developed by taking advantage of software techniques. The new QA platform can automatically calculate 3D dose distribution in the patient Computed tomography (CT) and report any plan issues detected from various items (e.g., target dose, dose‐volume histogram (DVH), and 3D gamma analysis).^13^, ^14^, ^15^, ^16^, ^17^, ^18^, ^19^ Because of these advantages, several institutes have used the software‐based QA platform as a primary‐ and secondary‐checking QA toolkit to evaluate IMRT plans.^20^ The Yonsei Cancer Center (YCC) is utilizing Mobius3D (Mobius Medical Systems) as a patient‐specific QA platform with an Elekta linear accelerator (LINAC).^21^ The Mobius3D platform was commissioned for a 6 MV X‐ray beam in our institute; its accurate assessment for clinical application was demonstrated in previous studies.^22^, ^23^

When a machine log file is received into the Mobius3D platform, the platform's fingerprinting system is performed to match the log file and its pertinent plan data. The fingerprinting system is well‐established for almost all cases; however, we suffered a specific problem during the last year of our application of the Mobius3D platform in practice. The problem is that the log file collected from the Elekta LINAC cannot find a corresponding plan even if the log data were correctly collected. This issue has twice been reported to Varian Medical Systems (case # 02495247, and case # 02727327), but an effective solution has not been presented. Currently, the vendor recommends re‐delivery of the plan to temporarily avoid this issue.

In this study, the cause of the mismatch between the Mobius3D platform (simply referred to as “Mobius3D” in the remainder of this paper) and the log file from the Elekta LINAC was analyzed using a patient‐specific QA case that was not recognized by Mobius3D. The results of our analysis using the re‐delivered data were compared with the modified data from the case that was mismatched. We presented a method for reducing the mismatching issue between the two systems.

## 2 MATERIALS AND METHODS

### 2.1 Communication between Mobius3D and th**e Elekta LINAC**

#### 2.1.1 Introduction of Mobius3D

Mobius3D is a platform for patient‐specific QA using a patient CT dataset, independent beam models, and a simplified collapsed‐cone dose calculation algorithm. Mobius3D is divided into two parts: MobiusCalc and MobiusFX.

MobiusCalc is a dose recalculation module based on an independent beam model and planned data extracted by the primary treatment planning system (TPS). In this module, the planned data are assessed using several items, such as the target dose coverage, DVH limits, 3D gamma comparison, and deliverability confirmation, which are then compared with machine delivery parameters. In Mobius3D, the MobiusCalc procedure is termed as “Plan Check.”

MobiusFX is another dose recalculation module accompanying MobiusCalc; the difference between the two modules lies only in whether the dose calculation procedure employs collected data from a LINAC machine (a.k.a. log data) or the planned data. In Mobius3D, the MobiusFX procedure is termed as “QA Check.” The collected log data from the LINAC machine are described in Section 2.1.2.

#### 2.1.2 Log file collected from the LINAC

**FIGURE 1:**
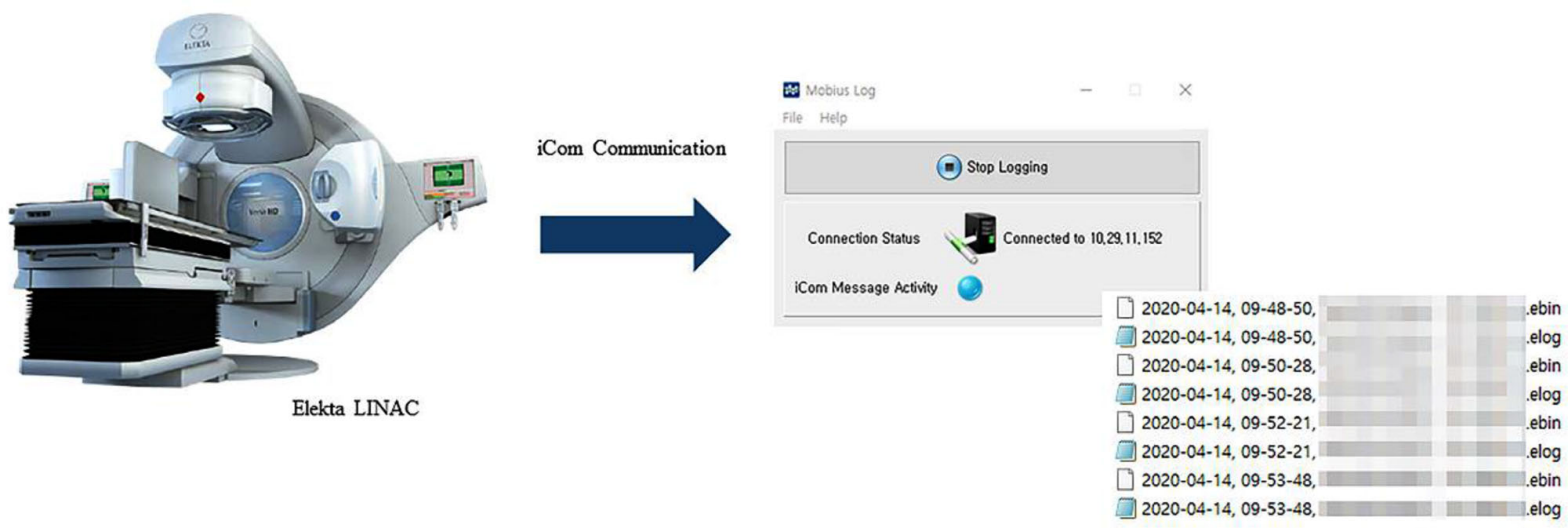
Schematic of the workflow for a Mobius3D acquisition from log files

**FIGURE 2:**
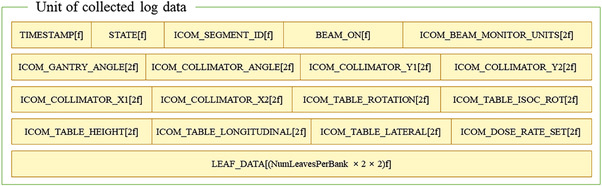
Structure of the log file generated by the Mobius Log Program, where “f” indicates a floating size

In an Elekta LINAC, the machine parameter data are extracted via iCom communication, and the data from the LINAC are collected and transferred to the Mobius3D system using Mobius Log, which is a program provided by the vendor. The schematic workflow for the collection of log data using Mobius Log is shown in Figure 1. The collected data per beam were saved in two files (*.ebin, and *.elog), and the contents were written in a combination of ASCII and binary format. The elog extension file contains a summary text of the treatment parameter information, and the ebin extension file contains the main performance data for the device.^24^ The structure of the ebin extension file is shown in Figure 2. Note that the structure is represented in the summary file (i.e., *.elog), and in the figure, the “f” indicates a floating size (i.e., 4 bytes).

### 2.2 Mismatching issue **between Mobius3D and the log file in an Elekta machine**

#### 2.2.1 Tolerances of beam fingerprinting in Mobius3D

The installed Mobius3D platform may receive several DICOM datasets from the TPS and several log files from the Mobius log program. To match the corresponding data between a DICOM dataset and its log data, Mobius3D provides a fingerprinting system; in other words, the QA check is performed using “passed” log data from the fingerprinting system that lie within their range of uncertainty. The conditions imposed on the fingerprinting system are listed below^24^:

1. Energy–must be equal to that of planned data
2. Initial X Jaw extents–within 1.1 cm of planned data (ignored for Elekta)
3. Initial Y Jaw extents–within 1.1 cm of planned data
4. Initial Gantry angle–within 4.1° of planned data
5. Initial collimator angle–within 4.1° of planned data
6. Initial MLC positions–within the field, while allowing a 1‐cm tolerance of planned data

#### 2.2.2 MLC conflicting error

**FIGURE 3:**
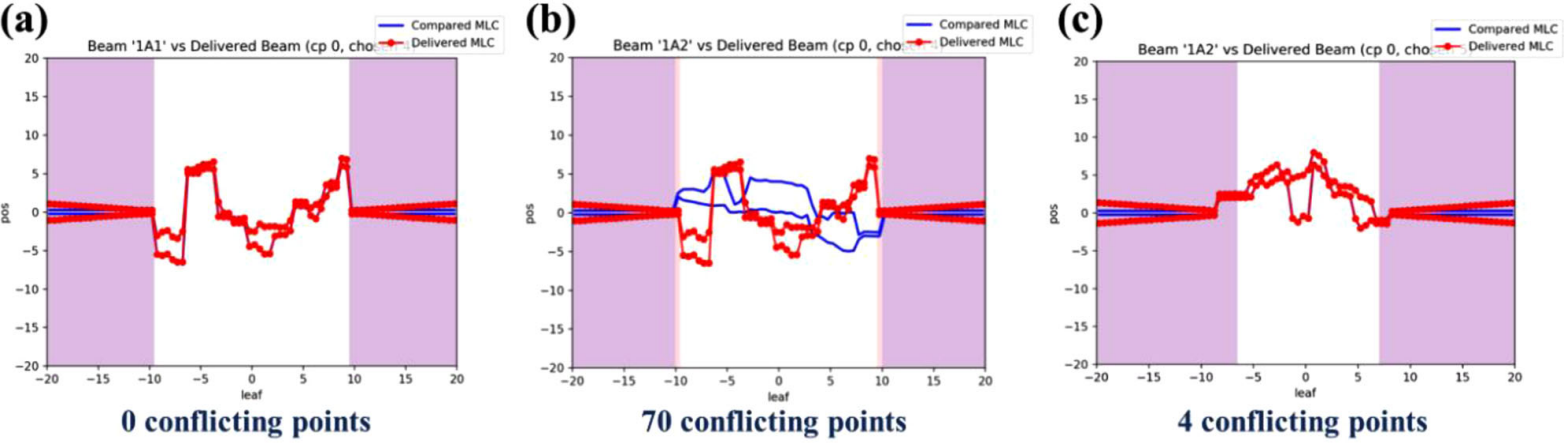
Example screenshots of multileaf collimator (MLC) comparisons between planned (red color) and logged data (blue line). There are three cases of MLC position comparison that appear as below: (a) matched MLC positions, (b) mismatched MLC positions with conflicting errors between different beams, (c) mismatched MLC positions with conflicting errors even though visually same such as (a). Note that the comparison results were generated by Mobius3D platform automatically

The MLC conflicting issue occurs frequently when using Mobius3D with an Elekta LINAC, owing to the differences in the initial MLC positions over the 1‐cm tolerance of the fingerprinting system, even if the initial MLC positions are virtually the same for the planned and logged data. The three different types of Mobius3D visualized analyses for checking the initial MLC positions are shown in Figure 3. A matched case (Figure 3a), a mismatched case caused by the differences between the beams (Figure 3b), and a mismatched case detected even when the initial MLC positions for the planned and delivered beams are virtually identical (Figure 3c). Visually, no conflicting point seems to be detected in Mobius3D.

### 2.3 Analysis of the MLC conflicting issue

To analyze the cause of the MLC conflicting issue, five mismatched cases were collected. Figure 3c shows an example case of MLC conflicting issue accrued in Mobius3D. Subsequently, the dose distribution was calculated using (1) TPS, (2) MobiusCalc (called M3D), and (3) MobiusFX. As mentioned before, the dose distribution determined using MobiusFX could not be directly calculated from the mismatched data. The detailed characteristics for all plans are shown in Table 1.^25^ The number of collected conflicting error points per case was 6, 4, 3, 3, and 12, and it is decided in the difference between initial and next MLC positions depending on each plan.

**TABLE 1:** Characteristics for five plans used in this study

Plan	Site	Number ofarc	Type ofarc*	Control points	Modulation index
1	Lymphoma	3	F	180/180	8.208
2	Lung	4	P	74/74/61/48	3.805
3	Lung	2	F	90/90	9.303
4	Lung	2	F	121/121	7.091
5	Liver	2	F	120/120	7.067

*Note*:^*^F, full arc; P: partial arc.

**FIGURE 4:**
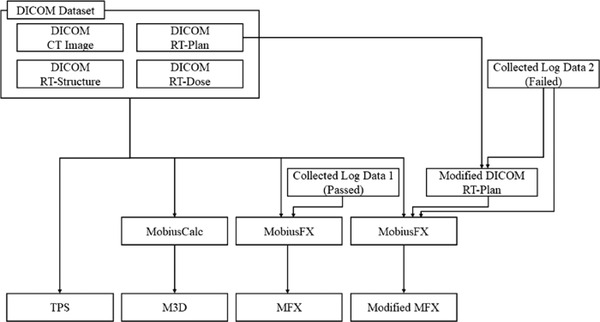
Schematic of the workflow for the analysis procedure

**FIGURE 5:**
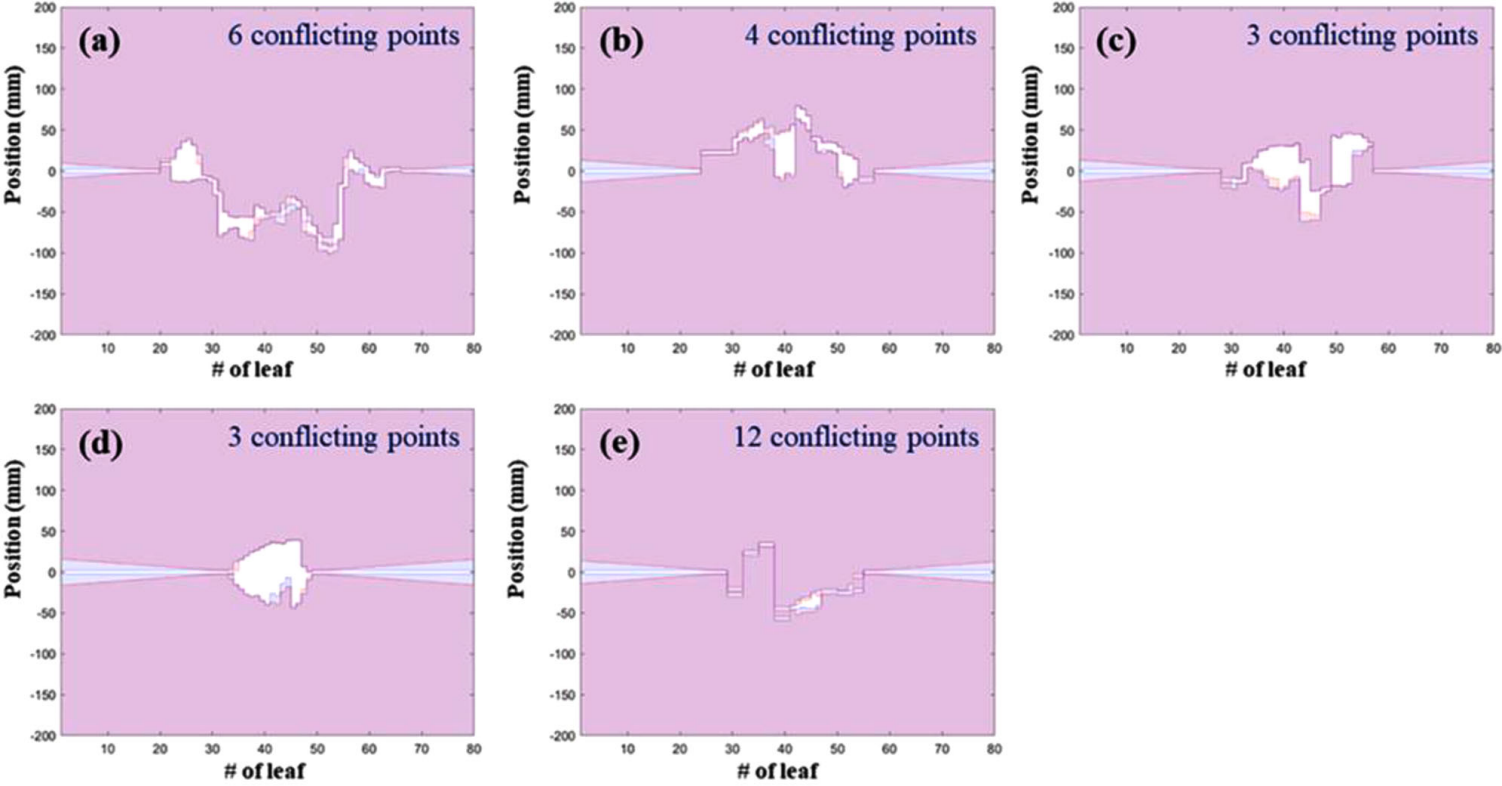
Overlapped images of initial MLC positions between RT‐Plan and logged data: (a) Plan 1, (b) Plan 2, (c) Plan 3, (d) Plan 4, and (e) Plan 5

Unfortunately, the fundamental reasons for the MLC conflicting issue could not be discovered without the Mobius3D source code. As two indirect methods, in this study, the comparison study was designed as a below: (1) re‐delivery and acquisition of new log data that passed the fingerprinting system (called MFX), and (2) adjustment of the initial MLC positions included in the DICOM RT‐Plan to pertinent positions within the 1‐cm tolerance range (called Modified MFX). Note that log file modification is also an option but not considered in this study owing to their own binary formats. The DICOM RT‐Plan was modified using MATLAB (The MathWorks) for each plan. The schematic workflow for calculating four different dose distributions using TPS, M3D, MFX, and Modified MFX is shown in Figure 4. Notably, the mismatched cases were collected from three Elekta Infinity linear accelerators (Elekta AB) with Agility MLCs. For the dose calculations, RayStation version 5.0.3.17 (RaySearch Laboratories) and Mobius3D 2.3 were utilized for the TPS and other Mobius‐related methods, respectively. Figure 5 shows the overlapped images of the initial MLC positions between the planned and the logged data in all cases.

From the calculated results, point dose and gamma index (GI) analyses were performed, and the results were compared with those of the TPS. With respect to the point dose, a specific volume with a size of an A1SL ionization chamber (Standard Imaging; i.e., 0.053 cc) in the planning target volume was defined for five DICOM datasets, and all the average values pertaining to each of the volumes were compared with each other. The dose errors were calculated according to Equation (1), where the actual dose value denotes the value calculated with the Mobius3D software (i.e., M3D, MFX, and modified MFX), and the planned dose value represents the calculated TPS dose value obtained using the Mobius3D software.

(1) $$\mathrm{Dose}\;\mathrm{Error}\;\left(\%\right)=\frac{\left(Actual\;Dose\;Value\left(Gy\right)-Planned\;Dose\;Value\left(Gy\right)\right)}{Planned\;Dose\;Value\left(Gy\right)}.$$

With respect to the GI analysis, the GI passing rate for 3D dose distribution was calculated. The action level of analyses was set to a 5% difference for the point dose. The GI was evaluated according to the pass rate calculated using the gamma evaluation method with a 3% dose difference and 3‐mm distance‐to‐agreement criteria.

In addition, the root‐mean‐square (RMS) errors for the MLC position between the plan and log data values were calculated to quantitatively evaluate the differences between the failed and passed log data. The failed log data originated from a comparison with the modified RT‐Plan in our study, and the passed log data originated from a comparison with the unmodified RT‐Plan. The RMS error was calculated at all control points and the tolerance was set to 0.5 mm. All the comparison results included a statistical analysis performed using a paired Student's *t*‐test from the SPSS 18.0 software package (SPSS, Inc.). Data were compared as follows: null hypothesis: H0 = 0 (no difference).

## 3 RESULTS

The point dose results obtained from M3D, MFX, and Modified MFX for the five QA cases are displayed in Table 2. All the results were within the tolerance defined in this study (i.e., 5%). The probability that there is no statistically significant difference between these results is greater than 0.999.

**TABLE 2:** Point dose results of the M3D, MFX (re‐delivery), and Modified MFX (modified RT‐Plan). The action level in our study was set to a 5% difference for point dose

	Point dose error (%)		
Plan number	M3D	MFX	Modified MFX
1	–1.6	–2.3	–0.3
2	2.1	1.8	1.3
3	2.5	3.0	2.5
4	2.4	2.2	2.2
5	1.0	0.0	0.0

The GI passing rates of the results calculated by M3D, MFX, and Modified MFX were compared with those calculated by the TPS and are displayed in Table 3. All the GI passing rates were higher than the tolerance for the action level ( > 90%). From the statistical results, the *p*‐value (*p* = 0.840) was not rejected; that is, it was confirmed that there was no significant difference between the passed and failed log data. As a result of comparing the RMS values for the MFX and the modified MFX, which were evaluated to confirm the positioning of the MLC, it was confirmed that the RMS values lay within 0.5 mm of the MLC position, and the difference of the maximum RMS values was 0.08 mm, which implies that the values were numerically similar. The results of the statistical analysis indicated that there was no statistically significant difference between the RMS values (*p* = 0.972).

**TABLE 3:** Passing rate of the gamma index (GI) analysis for the M3D, MFX (re‐delivery), and Modified MFX (modified RT‐Plan). The GI analysis was set to 3%/3 mm criteria

	GI passing rate (%)		
Plan number	M3D	MFX	Modified MFX
1	93.3	92.6	94.0
2	96.8	97.6	97.9
3	98.5	98.4	98.4
4	99.9	99.9	100.0
5	99.9	99.9	99.9

## 4 DISCUSSION

We performed an analysis to determine the cause of the MLC conflicting error that occurs when Mobius3D fingerprinting is applied in conjunction with an Elekta LINAC, even if the planned and delivered beams are virtually identical. Five mismatched cases were collected and analyzed for identifying the differences in the MLC positions and their related dose distributions, calculated using various methods. Our results confirmed that there was no significant difference between the passed and failed log data; nevertheless, the failed log data was filtered by the fingerprinting system of Mobius3D. This means that the visualization in Mobius3D is only visible between the position of the first collected log data and the RT‐Plan, but the initial position of all log data (i.e., ICOM_SEGMENT_ ID = 1) is verified by the fingerprinting system.

**FIGURE 6:**
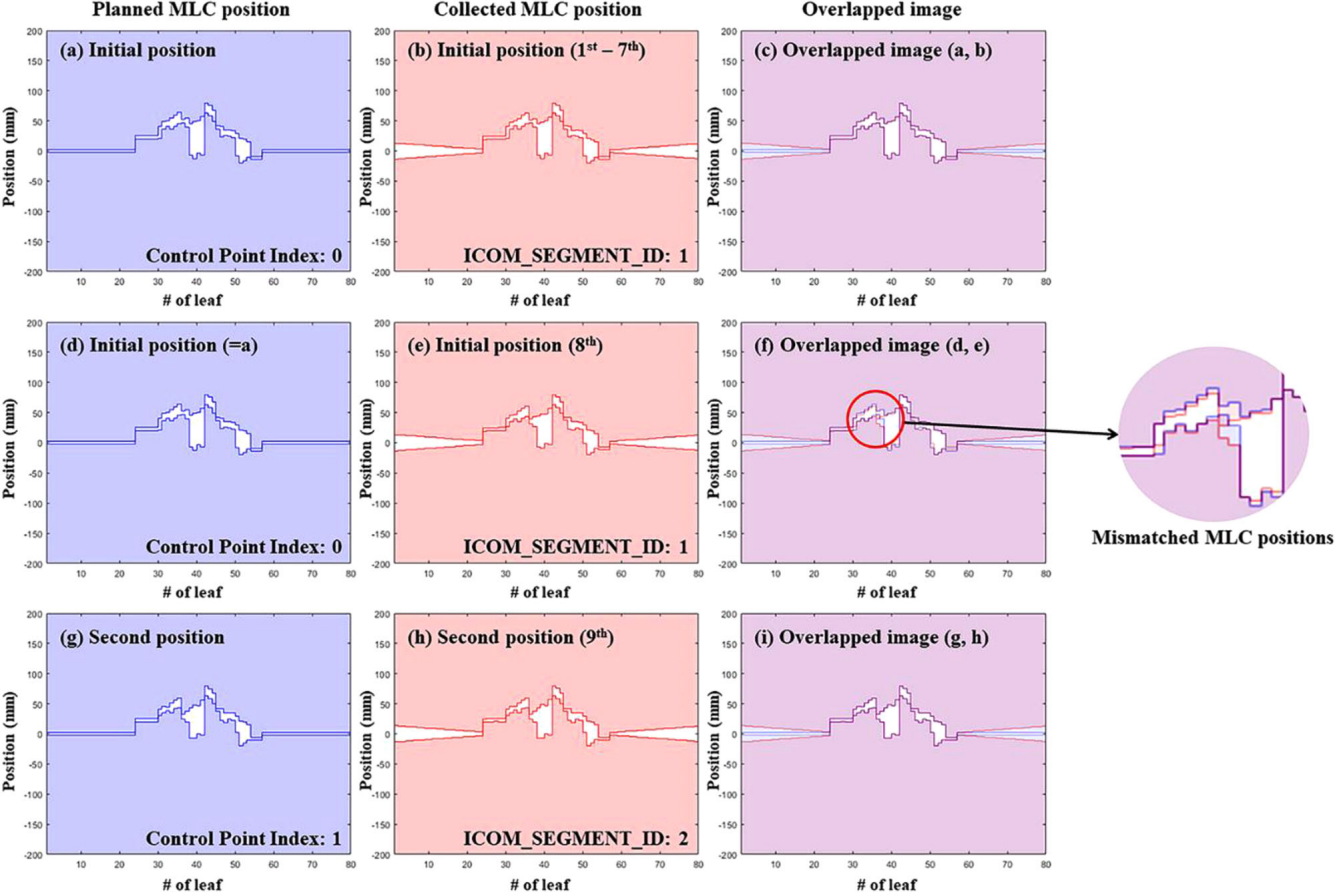
Example of the results of an MLC conflicting error due to the beam matched analysis method of the Mobius3D program. The set of the MLC positions, which are slightly altered toward the subsequent set of MLC positions, can be seen to deviate from the initial positions (e). The mismatched MLC positions are marked with a red circle (f). The number written as the ordinal number is the number of lines that acquire the MLC position every 0.3 s in Mobius3D program

This outcome can be attributed to the difference of an initial MLC position between a trigged log information and a planned data. To the best of our knowledge, the log information with respect to the MLC positions for Elekta machines was collected every approximately 0.3 s with a value of control point index. In this study, we found that the value of the initial point index might not change even when the MLC position moves according to the next index in all plans. At this point, the fingerprinting system can detect an MLC conflicting error if the logged initial MLC position (truly not the initial MLC position) is collected by more than 1‐cm tolerance of the planned data. Notably, the MLC conflicting errors could be caused due to the fast movement of MLCs between the initial and subsequent positions. An example of MLC position changes according to the collected MLC positions in a log file is shown in Figure 6. In Figure 6, a total of nine sets of MLC positions collected from log data were illustrated: logged data of first to eight (Figure 6b,e) for a planned data of initial position (Figure 6a,d), and logged data of ninth MLC positions (Figure 6g) for a planned data of second position (Figure 6h). As shown in Figure 6c,i, the images between planned and collected data were virtually identical within fingerprinting tolerance of Mobius3D system. In Figure 6f, however, the mismatched MLC positions were represented because the MLC position was collected during a transient period between initial and second positions. The ICOM_SEGMENT_ID during the transition period could depend on the collected timing of iCom communication, but unfortunately, in this case, the tagged ICOM_SEGMENT_ID for eight MLC position was 1 (i.e., initial position).

In addition, an RMS calculation was used to compare the set of the RT‐Plan of the TPS with the passed logs of the set of modified RT‐Plans and the failed logs. The values of the two results were found to be similar. The reason for this was that only the initial point changed. However, the RMS values modify the RT‐Plan of the TPS, implying that even larger differences may appear. Nevertheless, we found no significant difference since the modified method itself was adjusted to less than 1 cm, the MLC matching tolerance.

Two additional cases were excluded from this study: the pertinent positions for the modified MFX calculation that could not be found; that is, the cases in which the difference between the initial and the next MLC position was higher than twice the fingerprinting tolerance (i.e., 2 cm). These cases were successfully passed from the assessment of the conventional patient‐specific QA.

With respect to the frequency of occurrence in the study conducted in our institution, the MLC conflicting error was detected approximately 3% of the time. The QA procedure was re‐performed at such occurrences, following the vendor's recommendation.

## 5 CONCLUSION

We analyzed the causes of log file errors that may occur in Mobius3D software and Elekta machine log files and analyzed the dose errors that may be caused by incorrect log files by modifying the RT‐Plan file of patient‐specific QA. As a result, we confirmed that the dose difference was negligible, and we conclude that the problem can be resolved by using a simple modification of the fingerprinting system, such as a correct log data collection corresponding to a change of control point index. In the near future, we expect that vendors will be able to resolve the frequent MLC conflicting issues using the Elekta LINAC machine. Consequently, we anticipate that users’’ time and effort (e.g., a re‐delivery plan) expended in addressing this problem will be significantly reduced with future updates of Mobius3D.

## CONFLICT OF INTEREST

The authors have no conflict of interest to declare.

## AUTHOR CONTRIBUTION

Min Cheol Han and Tae‐Suk Suh conceived the presented idea. Yu‐Yun Noh and Han‐Back Shin carried out the experiment and analyzed the data. Min Cheol Han, Jihun Kim, and Yu‐Yun Noh designed the model, and Yu‐ Yun Noh and Min Cheol Han wrote the manuscript consultation with Jin Sung Kim and Tae‐Suk Suh. All authors provided critical feedback and helped shape the research, analysis, and manuscript.
